# Chondrosarcoma of the Skull Base: A Case Study and Literature Review

**DOI:** 10.7759/cureus.12412

**Published:** 2020-12-31

**Authors:** Anton Konovalov, Oleg Shekhtman, Anastasia P Shekhtman, Tatyana Bezborodova

**Affiliations:** 1 Vascular Surgery, Burdenko Neurosurgical Center, Moscow, RUS; 2 Vascular Surgery, Burdenko National Medical Research Center for Neurosurgery, Moscow, RUS; 3 Pathology, Russian Children's Clinical Hospital, Moscow, RUS; 4 Neurooncology, Burdenko Neurosurgical Center, Moscow, RUS

**Keywords:** chondrosarcoma, skull base tumors, surgical treatment, embryology

## Abstract

Chondrosarcomas (CSs) are rare malignant tumors composed of cells derived from the transformed chondrocytes. Only 2% of the total cases of CS are found at the skull base, thus representing a 0.1-0.2% prevalence. We present the case of a patient with CS at the middle cranial fossa who was admitted for surgery to the Burdenko National Medical Research Center of Neurosurgery. In addition, we engage in a review of the literature to discuss the current approaches to the diagnostics and surgery of CS and delve deep into its embryo- and oncogenesis.

## Introduction

Chondrosarcomas (CSs) are rare malignant mesenchymal tumors comprising cells derived from the transformed chondrocytes. CSs account for approximately 20% of all cases of skeletal system cancer. This is a heterogeneous group of tumors with various morphological features and clinical symptoms. CSs usually occur in the pelvic bone, shoulders, or along the superior metaphysical and diaphyseal regions of the appendicular skeleton. Intracranial CSs are rare lesions that are diagnosed in one in 1,000 cases of intracranial tumors [1]. Only 2% of all CSs are found at the skull base, thus accounting for a 0.1-0.2% prevalence [2,3]. They are characterized by slow growth and destruction of the basilar skull bones; however, these tumors belong to high-grade malignancies with rapid growth and early metastatic spread.

In this report, we discuss the case of a patient with CS at the middle cranial fossa who was admitted for surgery to the Burdenko National Medical Research Center of Neurosurgery. Additionally, we provide a review of the literature to elaborate on the current approaches to the diagnostics and surgery of CS and thoroughly explore its embryo- and oncogenesis.

## Case presentation

**Figure 1:**
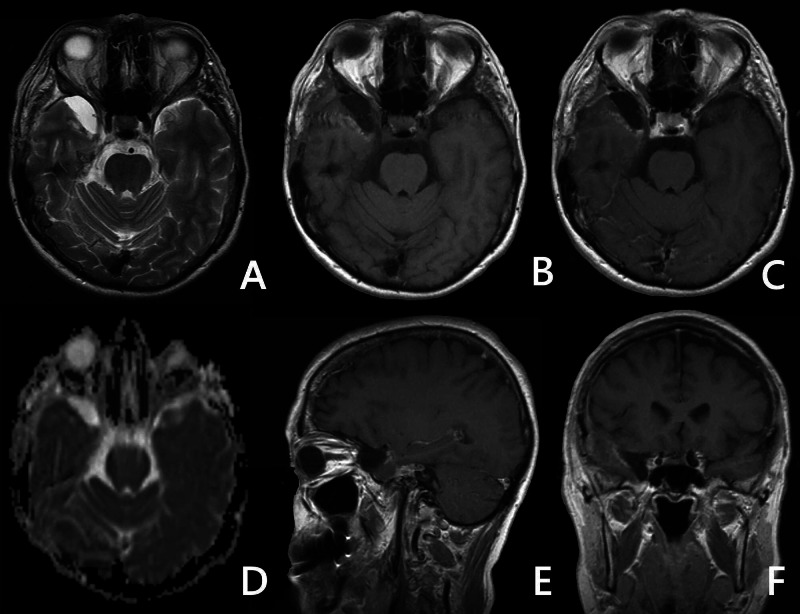
Preoperative MRI study (T2, T1, and T1 + C, DWI) A, B, C: the space-occupying lesion of the anterior sections of the middle cranial fossa in the region of the right temporal pole was determined. The lesion had a hyperintense signal at T2, and a hypointense signal at T1 compared to the brain tissue; no contrast uptake was noted. D: DWI shows no restriction. E, F: sagittal and coronal T1+C shows no contrast enhancement MRI: magnetic resonance imaging; DWI; diffusion-weighted imaging

Our patient was a 54-year-old female who had initially approached a local neurologist with recurrent unilateral headaches in the right temporal region, which had been effectively controlled by non-steroidal anti-inflammatory drugs. An MRI had been performed one year ago after the patient experienced a single generalized clonic seizure. At imaging, a compact, well-delineated lesion of the medial temporal area was found. It had a hyperintense signal in T2 and a hypointense signal at T1 and showed no contrast uptake. Further study demonstrated that the tumor was situated extradurally and destructed petrosal crest spreading along the petroclival joint into the posterior cranial fossa. Thus, a mesenchymal tumor of the middles fossa was suspected (Figure 1). Further consultation with the Tumor Board confirmed our decision for surgical removal.

Surgery

**Figure 2:**
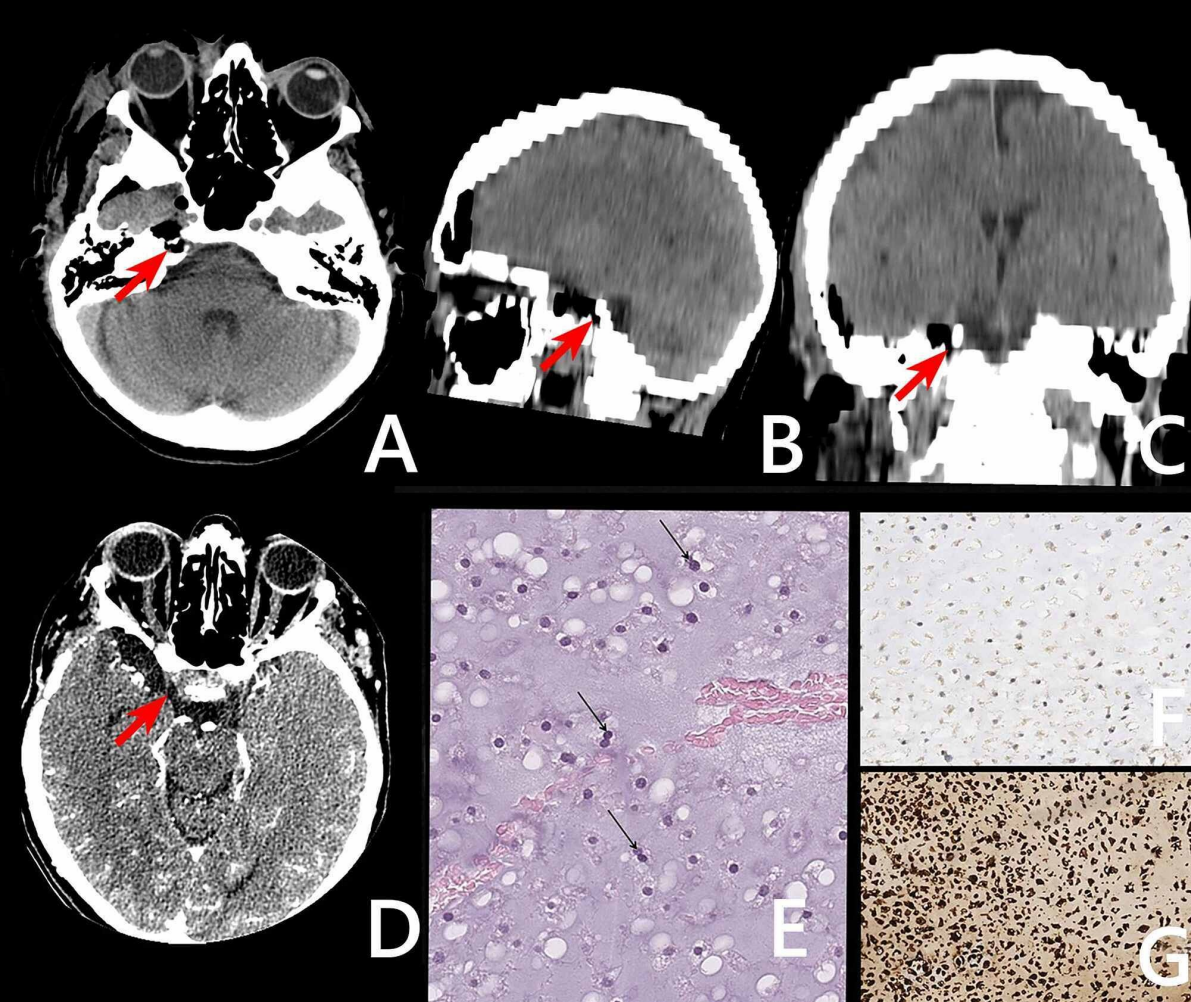
Postoperative brain CT and histology A, B, C, D: postoperative brain CT scan (with and without contrast enhancement); the postoperative changes in the area of the right temporal pole and medial parts of the middle cranial fossa were determined intradurally; the red arrow shows the petroclival junction tumor bed. E: staining: hematoxylin and eosin, x400. A large number of binuclear cells were observed. Mitotic activity was undetectable. F: neoplastic cells were negative for brachyury. G: a positive immunohistochemical reaction with an S100 protein antibody was determined in the tumor sample CT: computed tomography

Surgery was performed via the pterional approach. Opticocarotid cistern was opened widely to release cerebrospinal fluid (CSF). Sylvian fissure was then dissected to approach the medial temporal region. Large extradural mass separated with thinned dura was visualized. Once opened, a very soft, grey-colored gelatinous-like density tumor was observed. Its soft part was easily removed with ultrasonic aspiration. Moving further, the posterior tumor became denser with osseous inclusions. We managed to break apart and remove some of them. Large ones were left in their place. The basilar pyramid and sphenoid bone were partly destroyed. Tumor volume was resected. Hemostasis was achieved with oxidized cellulose and hemostatic gelatin matrix. The bone flap was kept in place and fixed, and the wound was closed in layers. The patient was shifted to the ward. The postoperative course was uneventful, and the follow-up brain CT (Figure 2) showed postoperative changes.

Pathology report

Abundant grey cartilage matrix production was identified. Irregularly shaped lobules of cartilage varying in size and shape were present. Fibrous bands separated these lobules. The chondrocytes were atypical, with variable size and shape, and contained enlarged hyperchromatic nuclei. Binucleation and chondroid matrix liquefaction were seen. The immunohistochemical examination was performed with the antibodies to S100, brachyury, epithelial membrane antigen (EMA), and Ki67. The expression of S100 was revealed in the tumor tissue, and the Ki 67 proliferative activity was about 2%. No reactions with antibodies to brachyury and EMA were found in the tumor tissue within the material under study. Thus, the final diagnosis was formulated as an atypical cartilaginous tumor/chondrosarcoma grade I (Figure 2); the International Classification of Diseases for Oncology (ICD-O) code: 9220/1 (ICD D48.0).

The patient was discharged on the seventh day after surgery in satisfactory condition. The follow-up brain MRI showed no signs of tumor progression after three, six, and 12 months. After consulting an oncologist, we decided to adhere to the expectant treatment and refrain from adjuvant treatment.

## Discussion

Epidemiology

Intracranial CSs are extremely rare basilar skull tumors that account for 0.16% of all intracranial tumors and about 6% of all basilar skull tumors [4]. Only 2% of all CSs affect skull bones [2,3]. Clinical symptoms typically occur at the age of 40-70 years. Most of the cases are sporadic, and no risk factors for the CS formation have been identified so far [5]. CSs could be found as de novo lesions or be associated with very rare skeletal disorders such as Ollier disease, Maffucci syndrome, Paget’s disease, and osteochondroma [4].

Clinical findings largely depend on the location and size of the tumor expansion. Patients usually complain of headaches, diplopia, entotic sound, and hearing impairment, which sometimes may be accompanied by decreased sensitivity in trigeminal zones, and cerebellar and brain stem symptoms [6,7]. Asymptomatic cases when CS is accidentally found on MRI have also been described.

CS is one of the primary malignant tumors of bone tissue originating from chondrocytes. Most CSs belong to the low grade (I-II), which are malignancies with slow growth and a low incidence of metastatic spread [4]. Typically, intracranial CSs occur in the paraclival region, developing from the sphenopetrosal and petroclival synchondrosis [8]. However, rare cases of CSs derived from the pluripotent cells of the cerebral meninges, parenchyma, or vascular plexus have also been described [9]. The emergence of CS at synchondrosis junctions can be attributed to the embryology of the skull bone formation.

Embryogenesis and ossification mechanisms

Two bone-shaping mechanisms have been described so far: intramembranous and endochondral ossification. The flat bones of the cranial vault and facial skull are formed by intramembranous ossification, i.e., directly based on fetal mesenchyme. Endochondral ossification comes from the neural crest cells that, through the cartilaginous stage, lead to the formation of basilar skull bones: ethmoid bone, sphenoid bone, and intraparietal part (below the superior nuchal line) of the occipital bone [6]. The coronary suture between the frontal and parietal bones is the border between two precursors that are used as a basis for the skull bone formation: the fetal mesenchyme and the neural crest cells.

**Figure 3:**
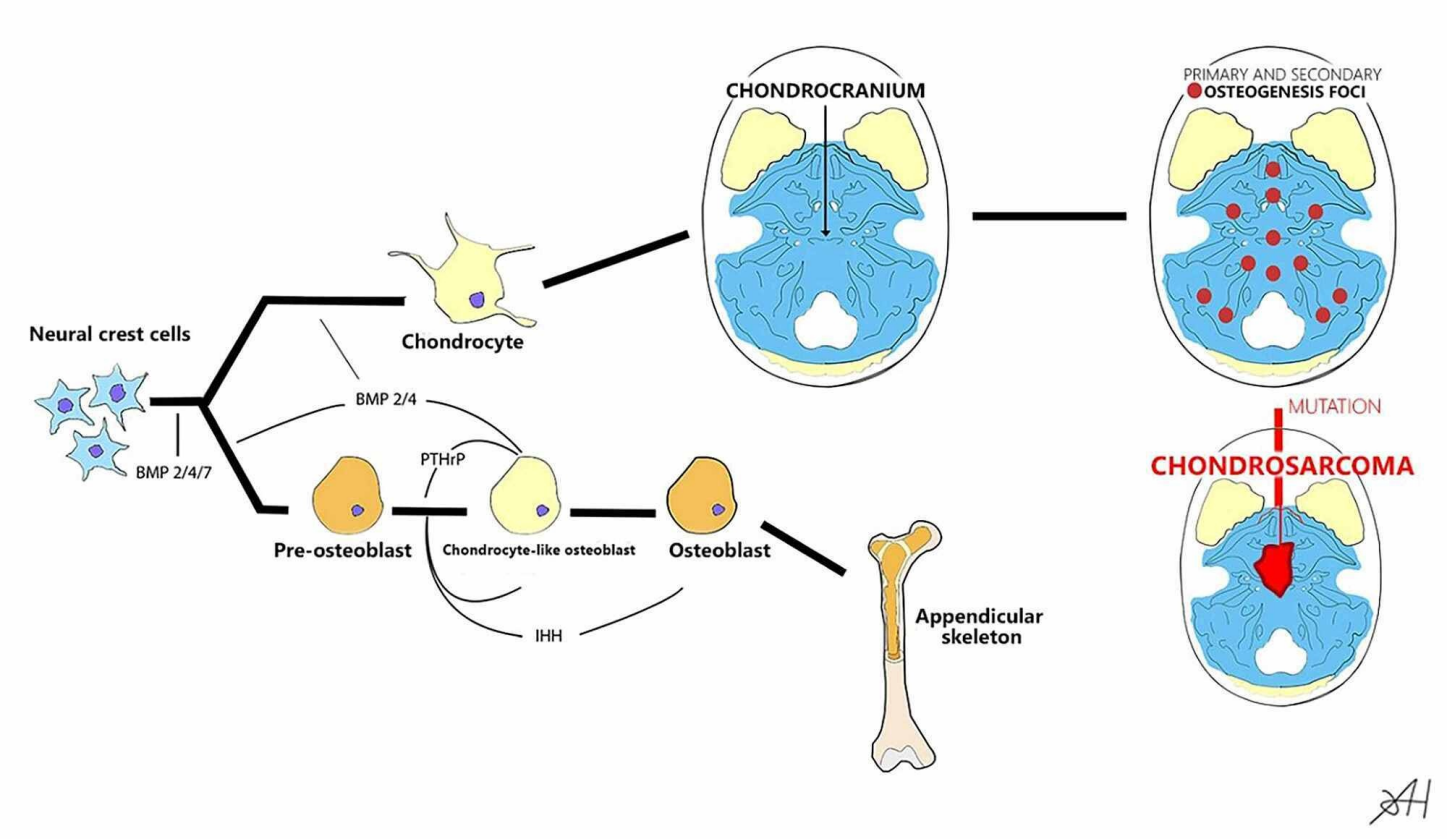
Chondrosarcoma oncogenesis Formation processes of chondrocytes and osteoblasts with subsequent ossification options: endochondral ossification of the chondrocranium and intramembranous ossification of the appendicular skeleton BMP: bone morphogenetic proteins; PTHrP: parathyroid hormone-related protein; IHH: Indian hedgehog

In intramembranous ossification, bone development occurs directly from the mesenchyme. Mesenchymal cells originating from the neural crest proliferate and differentiate into osteoprogenitor cells. As the differentiation progresses, the shape and size of cells change, and cytoplasm volume and amount of granular endoplasmic reticulum are increased, leading to the cells becoming osteoblasts. The osteoblasts secrete a collagen-proteoglycan matrix (mainly type I collagen); this way, the extracellular matrix binds calcium salts to becomes calcified. Osteoblasts are separated from the calcification area by a layer of osteoid matrix. When osteoblasts enter the calcified osteoid matrix, they become osteocytes [6]. The chondrosarcoma oncogenesis is depicted in Figure 3.

The bone morphogenetic proteins (BMP) and activation of the transcription factor CBFA1 (Runx2) play an important role in the intramembranous ossification mechanism. The BMPs (BMP2, BMP4, and BMP7) are believed to activate CBFA1 (Runx2). The CBFA1 factor, in turn, triggers the differentiation of neural crest mesenchyme cells directly into the osteoblasts. The transcription factor CBFA1 (Runx2) also upregulates the expression of type I collagen, osteocalcin, osteopontin, and other specific proteins for the bone extracellular matrix formation [6].

Endochondral ossification is the bone formation process indirectly through the cartilaginous stage. This process includes two stages, namely the formation of a primary and secondary ossification or osteogenesis foci.

Formation of the primary ossification center begins with the condensation of mesenchymal progenitor cells at the sites of the future bone formation. The cells in the central lesion part divide and increase in size. Unlike intramembranous ossification, the cells are differentiated into the chondrocytes rather than osteoblasts at this stage. Chondrocytes begin to produce an extracellular matrix rich in type II and X collagen and specific proteoglycans. Thus, a cartilaginous model of the future bone is formed. Moreover, the cellular composition in the center and along the periphery of these foci differs from each other. In the center, the cells are larger, rounded, and located at some distance from each other due to the produced extracellular matrix. The process of chondrocyte division and differentiation in the central part of the cartilaginous model is called interstitial growth, while the periphery is occupied by the less differentiated osteoprogenitor cells forming the perichondrium (the future periosteum). This process is called appositional growth.

Subsequently, the cartilaginous invasion by the blood vessels begins. Hypertrophic chondrocytes in the central part are destroyed by apoptosis. The resulting space becomes the bone marrow. When the cartilaginous cells die, it stimulates the groups of osteoprogenitor cells to be differentiated into osteoblasts. The osteoblasts produce a mineralized bone matrix around the cartilaginous model of the future bone that serves as a scaffold and initiates the formation of the cortical bone plate. This is the process of a bone matrix formation on the partially destroyed cartilage by osteoblasts. Eventually, all cartilage is completely replaced by the bone.

Thus, in rare cases, some of the chondrocytes can persist for many years, which can lead to malignant transformation (Figure 3).

Morphology

During the surgery, CSs are typically the extradural space-occupying lesions that destroy the bone structure and have a jelly-like consistency [5]. In some cases, calcification of the surrounding tissue of the tumor may occur. Morphologically, CS is a broad group of tumors that typically contains the cartilaginous matrix produced by neoplastic cells. There are four histological subtypes of CSs: conventional or classical, mesenchymal, clear cell, and dedifferentiated. There are no clinically significant differences between the subtypes presented. Conventional chondrosarcoma is the predominant type for the cranial region. According to the WHO classification of head tumors, the types of classical CSs are as follows:

1. Classical CS grade I or atypical chondromatous tumor is the most common type of cranial CS (about 80% of cases) and shares most microscopic features with a benign cartilaginous tumor - chondroma [4]. Histologically, the grade I CS consists of benign-looking hyaline cartilage lobules with various shapes and sizes, with a low cellular density. Cytologically, the cells correspond to the normal chondrocytes and can be arranged in clusters; they are located in lacunae and contain scant cytoplasm. The cell nuclei are usually small, monomorphic, round, and hyperchromic due to densely packed chromatin. A few binuclear and stellate cells can be observed in CS grade I. Mitotic activity is very low: up to two mitotic figures per 10 high-power fields. Unlike chondroma, CS is characterized by the destruction of preexisting bone tissue, spreading into intratrabecular space.

2. Classical CSs grade II of the skull bones are less common (17.8% of cases - Bloch) [4] than grade I. CSs grade II demonstrate more prominent nuclear polymorphism and some myxoid changes of the extracellular matrix. Rare mitotic figures can be found: two or more figures per 10 high-power fields.

3. Classical CSs grade III account for only 1.8% of the total cases of cranial CSs [3]. These tumors consist of poorly differentiated cells with significant cellular and nuclear polymorphism and high mitotic activity. A more compact arrangement of neoplastic cells can be noted on the periphery of the lobules. The immunophenotype of classic CSs shows a positive reaction with S100 protein, regardless of the degree of malignancy. The IDH1 mutation can be observed in 30% of classic CSs. More often, IDH1/2 mutations are found in classic grade III CS (44%), and less often in grade II (39%) and grade I (21%) CSs. The morphological differential diagnosis includes the following entities: chordoma, chondromyxoid fibroma, chondromesenchymal hamartoma, chondroblastic osteosarcoma, and chondroma.

In most cases, CSs affect the clivus and spread to the middle cranial fossa around the sellar (30-50%) and to the posterior cranial fossa (50%) [10]. The widespread pattern of growth makes radical resection hard to complete. At present, the most effective treatment is a combination of surgical removal and fractionated radiation therapy. The close contact with the nervous and vascular structures of the basilar skull creates a significant risk of post-treatment neurological damage. The prognosis for the patients with intracranial CS depends on surgical excision, morphological subtype, and previous treatment (microsurgical or radiological).

Diagnostics

Imaging includes brain CT and MRI data. On axial CT, CSs demonstrate an isodense signal as the soft tissue lesion, often with the areas of deformation or destruction of the basilar skull bones. Contrast enhancing brings heterogeneous contrast uptake, but there are cases when CSs are non-enhanced (grade 1). In 50% of patients, CS carries calcifications, typically in the form of rings or half rings.

On MRIs, CSs are hypointense in the T1 mode with a weak heterogeneous contrast uptake. Some authors have determined a typical contrasting pattern in the form of a "spindle" or "honeycomb" [5]. In some cases, the contrast is not noted. Low-grade malignant tumors are poorly vascularized and weakly contrasted. In T2, CSs are hyperintense and, accordingly, poorly defined in the T2 fluid-attenuated inversion recovery (FLAIR) mode. Differential diagnosis includes the following: chordoma, chondromyxoid fibroma, cholesteatoma of the petrous apex, metastasis, plasmacytoma, and nasopharyngeal carcinoma [4].

Treatment and prognosis

The main CS treatment strategy consists of resection to the maximum possible degree, followed by radiation therapy. Surgery aims at both decompression of the neural structures (cranial nerves) and histology. Several clinical studies have demonstrated improved prognosis in surgically managed cases [11-13]. In 2014, Jones et al. compared the results of CS surgery with conservative treatment in a group of 226 patients. The 10-year survival rate was higher (69.3%) in the surgical treatment group compared to 54% in the conservative treatment group [8]. Cranial nerve deficits after surgery are rarely restored; moreover, morbidity risks are higher if resection is planned [2,11-13]. Thus, surgery remains controversial although most experts consider partial CS removal with subsequent radiation the optimal strategy [2,11-13].

**Table 1:** Details of recent case series with treatment results SRS: stereotactic radiosurgery

Author	Year	Number of patients	Treatment type (number of patients)	Results
Bloch et al. [3]	2009	560	Surgery (161), surgery + radiotherapy	5-year mortality rate is 25% for the first group and 9% for the second group
Jones et al. [7]	2014	226	Surgery (128), surgery and radiotherapy (81), radiotherapy (5), follow-up (12)	10-year survival rate of the patients with surgical treatment is 69.3%; for the patients without surgical treatment: 53.9%
Kano et al. [11]	2015	46	Surgery + SRS (36), radiotherapy + SRS (5), SRS (5)	5-year and 10-year survival rate without progression is 85% and 70%
Feuvret et al. [12]	2016	159	Surgery + proton beam therapy	Overall 5-year and 10-year survival rate is 94.9% and 87%
Carlson et al. [13]	2015	45	Surgery (15), surgery + radiotherapy (30)	5-year and 10-year survival rate without progression is 70% and 56%
Weber et al. [14]	2015	77	Proton beam therapy	Overall 8-year survival rate is 93.5%
Simon et al. [2]	2018	47	Surgery (24), surgery + proton beam therapy (23)	5-year and 10-year survival rate without progression for the first group is 68% and 52%; for the second group: 100% and 88%
Lu et al. [10]	2020	158	Surgery + radiotherapy	Overall 5-year and 10-year survival rate is 95% and 70%

Radiosurgery is recommended as the second stage in the postoperative treatment of patients with CSs. Several studies have demonstrated a significant effect of radiation on prognosis and survival rate [5,14]. Weber et al. showed that the eight-year survival rate after proton beam therapy was 93.5% [14]. However, radiosurgical treatment is not without risks. Complications include radiation damage to the cranial nerves, brain stem, large vessels (internal carotid artery), pituitary gland, etc. Proton beam therapy allows to plan treatment with high precision and reduce the dose impact to adjacent structures [14]. Unfortunately, proton beam therapy is not widely available and can be found in large centers only [11].

Unfortunately, we could not find any class I studies evaluating a combined CS treatment. Given the very slow growth of these tumors and their good response to radiosurgery, partial excision and radiation exposure represent the best strategy to avoid disabling neurologic deficits.

Chemotherapy for CSs has not demonstrated considerable benefits [4,10]. In some cases, chemotherapy is performed as a salvation option in patients in despair with grade III CS that has shown no response to combined treatment [5]. Further studies of CSs molecular biology and genetics may help to develop an effective targeted therapy for these rare tumors in the future.

## Conclusions

Intracranial CSs are rare skull base neoplasms that are challenging both in terms of diagnostics and surgical treatment. The current optimal strategy comprises maximal safe resection followed by radiosurgery based on the pathology pattern. When scheduling a combined therapy, priority should be given to the quality of life and preservation of neurological function (cranial nerves). Tumor biology requires further study in order to develop targeted chemotherapy options.
